# Universal digital high-resolution melt: a novel approach to broad-based profiling of heterogeneous biological samples

**DOI:** 10.1093/nar/gkt684

**Published:** 2013-08-09

**Authors:** Stephanie I. Fraley, Justin Hardick, Billie Jo Masek, Pornpat Athamanolap, Richard E. Rothman, Charlotte A. Gaydos, Karen C. Carroll, Teresa Wakefield, Tza-Huei Wang, Samuel Yang

**Affiliations:** ^1^Department of Biomedical Engineering, The Johns Hopkins University, Baltimore, MD 21218, USA, ^2^Department of Emergency Medicine, The Johns Hopkins University, Baltimore, MD 21218, USA, ^3^Division of Infectious Disease, Department of Medicine, The Johns Hopkins University, Baltimore, MD 21218, USA, ^4^Division of Medical Microbiology, Department of Pathology, The Johns Hopkins University, Baltimore, MD 21218, USA, ^5^The Johns Hopkins Hospital, Baltimore, MD 21287, USA and ^6^Department of Mechanical Engineering, The Johns Hopkins University, Baltimore, MD 21218, USA

## Abstract

Comprehensive profiling of nucleic acids in genetically heterogeneous samples is important for clinical and basic research applications. Universal digital high-resolution melt (U-dHRM) is a new approach to broad-based PCR diagnostics and profiling technologies that can overcome issues of poor sensitivity due to contaminating nucleic acids and poor specificity due to primer or probe hybridization inaccuracies for single nucleotide variations. The U-dHRM approach uses broad-based primers or ligated adapter sequences to universally amplify all nucleic acid molecules in a heterogeneous sample, which have been partitioned, as in digital PCR. Extensive assay optimization enables direct sequence identification by algorithm-based matching of melt curve shape and Tm to a database of known sequence-specific melt curves. We show that single-molecule detection and single nucleotide sensitivity is possible. The feasibility and utility of U-dHRM is demonstrated through detection of bacteria associated with polymicrobial blood infection and microRNAs (miRNAs) associated with host response to infection. U-dHRM using broad-based 16S rRNA gene primers demonstrates universal single cell detection of bacterial pathogens, even in the presence of larger amounts of contaminating bacteria; U-dHRM using universally adapted Lethal-7 miRNAs in a heterogeneous mixture showcases the single copy sensitivity and single nucleotide specificity of this approach.

## INTRODUCTION

A key challenge in the fields of clinical diagnostic development and basic biomolecular research involves the ability to quantitatively and accurately identify single copies of numerous nucleic acid targets of interest in a heterogeneous sample (1–6). Typically, hybridization assays such as microarrays are used for broad semi-quantitative profiling, whereas multi-reaction quantitative PCR (qPCR) is used for enumeration of multiple nucleic acid sequences (4). Both of these techniques lack the sensitivity to detect individual sequences at low level concentrations, as a sample must be split across many reactions containing distinct sets of primers or probes (7–9). Likewise, their multiplexed formats rely on highly specific primer or probe annealing to discriminate single nucleotide differences, often resulting in inaccuracies. Prior knowledge of target molecule sequences within a sample is required and discovery of novel species is not possible. With the advent of next-generation sequencing (NGS), highly sensitive, specific and multiplexed detection of both known and unknown target molecules in a mixed sample is possible, and NGS’s utility as a diagnostic and research tool is being explored. However, errors in base calling, alignment and assembly of sequence data occur (10); deep sequencing remains a costly, time-consuming and multi-step process, which limits accessibility for most clinical and basic research laboratories (11).

We have developed a novel technique, universal digital high-resolution melt (U-dHRM), which allows highly sensitive, specific and broad-based detection, as well as discovery of unanticipated nucleic acid genotypes in a single rapid assay that is less expensive and generally more accessible than NGS. This new technique unites and reinvents aspects of limiting dilution/digital PCR (12,13), broad-based/universal amplification (14), high-resolution melt (HRM) (15–18) and microfluidic theory, resulting in a practical genotyping technology that is highly promising for diagnostic applications. To demonstrate the utility of U-dHRM, we consider two applications: polymicrobial sepsis diagnosis and therapeutic monitoring of host microRNA (miRNA) involved in infection.

miRNAs are short (19–22 nt) non-coding RNAs that interact with messenger RNAs to regulate gene expression. Circulating miRNAs, released by cells into the blood, hold great promise as biomarkers of disease status and treatment efficacy (19–22). To our knowledge, the use of broad-based HRM to identify miRNAs has not been proposed. This is probably because broad-based bulk HRM of a mixture of miRNAs cannot resolve individual sequences. Several groups, including our own, have proposed broad-based bulk HRM methods for identification of bacterial species involved in sepsis (14,23–27). These methods typically use universal primers to amplify hypervariable regions of the 16S rRNA gene. Subsequently, HRM is performed using DNA saturating dyes (23,24) or multiple color probes (25,26) to generate melt signatures that uniquely identify specific bacterial species. Even though generally reproducible melt signatures are obtained when bacterial species are measured independently, they cannot be individually identified when multiple species are present in the sample simultaneously or when contaminating bacteria are present, as often occurs clinically (14).

Digitization addresses a critical and unique need in the advancement of HRM technology to overcome limitations on sensitivity. Recently, several groups have used HRM after oncogene-specific digital PCR to detect the low-level presence of mutations or methylation of a gene of interest (28–31). Here, we address remaining limitations of HRM by developing U-dHRM, which allows broad-based detection of numerous targets and enables specific sequence identification by database matching. U-dHRM for pathogen detection is not hindered by contamination from environmental microbes, contamination within PCR reagents or multi-species polymicrobial infections, which have to this point proven extremely problematic to bulk HRM assays as well as qPCR and culture-based diagnostic assays (27,32–35). The potential of U-dHRM to quantify single molecules in mixed samples may also, as semi-quantitative studies suggest, further improve clinical diagnosis by helping distinguish whether a microbe is a pathogen or contaminant in instances where it could be either (33). Likewise, U-dHRM for miRNA profiling is not limited to a certain number of miRNAs but potentiates genome-wide profiling and discovery with single-nucleotide specificity and single-molecule sensitivity.

Herein, as a proof of concept, we demonstrate application of the U-dHRM approach for broad-based detection in heterogeneous samples containing multiple pathogens and contaminants commonly involved in polymicrobial sepsis (2) and broad-based detection of all members of the Lethal-7 (Let-7) family of closely related host miRNAs known to be key infection-related biomarkers, which are difficult to resolve by other profiling methods (20).

## MATERIALS AND METHODS

### miRNA universal tag design

Multiple short tag sequences were generated following basic primer design rules. Each tag pair was entered into NCBI-BLAST (36) to screen for homology with other human or bacterial sequences, then tested using OligoCalc (37) to determine the propensity for each oligo to form hairpins and self-anneal, as well as the degree of 3′ end complementarity. The tag primer sequences used for these experiments are: Tag-F (5′-CCATAGACGTAGCAACG ATCG-3′) and Tag-R (5′-GATGCAAGGACTATCCACTCAC-3′). For this study, tagged cDNA corresponding to the 10 miRNA sequences in Table 1 were synthesized by Integrated DNA technologies (IDT, Coralville, IA).

**Table 1.** Lethal-7 family and related miRNA sequences

Name	Sequence	Difference
let-7a	TGAGGTAGTAGGTTGTATAGTT	reference
let-7b	TGAGGTAGTAGGTTGT***G***T***G***GTT	2 nt
let-7c	TGAGGTAGTAGGTTGTAT***G***GTT	1 nt
let-7d	***A***GAGGTAGTAGGTTG***C***ATAGT	2 nt
let-7e	TGAGGTAG***G***AGGTTGTATAGT	1 nt
let-7f	TGAGGTAGTAG***A***TTGTATAGTT	1 nt
let-7g	TGAGGTAGTAG***T***TTGTA***C***AGT	2 nt
let-7i	TGAGGTAGTAG***T***TTGT***GCT***GT	4 nt
miR-98	TGAGGTAGTA***A***GTTGTAT***T***GTT	2 nt
miR-29	T***AGCACCA***T***CT***G***AAA***T***CGGT***T***A***	17 nt

Differences in nucleotide sequence are shown in bold type.

### Polymicrobial primer design

An alignment was performed using BioEdit (Ibis Biosciences, Carlsbad CA) on 16S rRNA gene sequences for the pathogens listed in Table 2. The V6 region was chosen to discriminate these pathogens based on uMELT (15) model predictions of amplicon melt curves. The V6 primers used were designed and validated previously by Yang *et al.* (14). Their sequences are V6-F (5′-GGAGCATGTGGTTTAATTCGA-3′) and V6-R (5′-AGCTGACGACANCCATGCA-3′). For this study, primers were synthesized by IDT.

**Table 2.** Clinically relevant bacteria involved in sepsis

Gram	Genus	Species	Common role in blood culture
+	*Staphylococcus*	*lugdunensis*	Contaminant/Emerging Pathogen
+	*Staphylococcus*	*aureus*	Pathogen
+	*Staphylococcus*	*saprophyticus*	Contaminant/Emerging Pathogen
+	*Staphylococcus*	*epidermidis*	Contaminant/Emerging Pathogen
+	*Streptococcus*	*agalactiae*	Pathogen
+	*Enterococcus*	*faecalis*	Pathogen
+	*Propionibacterium*	*acnes*	Contaminant
**−**	*Pseudomonas*	*aeruginosa*	Pathogen
**−**	*Klebsiella*	*pneumoniae*	Pathogen
**−**	*Salmonella*	*choleraesuis*	Pathogen
**−**	*Salmonella*	*enteritidis*	Pathogen
**−**	*Salmonella*	*dublin*	Pathogen

### Nucleic acid extraction and sequencing

DNA was extracted from clinically isolated or American Type Culture Collection (ATCC) acquired bacterial strains: *Staphylococcus lugdunensis, Salmonella enteritidis, Staphylococcus aureus, Salmonella choleraesuis, Staphylococcus saprophyticus, Staphylococcus epidermidis, Salmonella Dublin, Klebsiella pneumoniae, Enterococcus faecalis, Propionibacterium acnes, Pseudomonas aeruginosa, Streptococcus agalactiae)* using Roche MagNA Pure LC (Roche Diagnostics, Indianapolis, IN) with the DNA Isolation Kit I (Roche Diagnostics) using a 200 µl of input volume and a 100 µl of final elution volume per manufacturer’s instructions. Sanger sequencing was performed post-PCR and post-HRM. First ExoSAP-IT PCR clean-up kit (Affymetrix, Santa Clara, CA) was used as directed, and then samples were processed by the Johns Hopkins Genetic Resources Core Facility.

### U-dHRM reaction design

*General U-dHRM optimization.* Primer concentration, annealing temperature and ammonium and potassium concentration (38) were all optimized by evaluating various conditions until reactions could be performed to reliably amplify single copies of template and give reproducible U-dHRM curves. The incorporation of temperature calibrator sequences to the reactions was performed and optimized to overcome well-to-well and experiment-to-experiment variations in heating during melt curve generation. Temperature calibrator duplexes, blocked to amplification by 3′ end modification, were described previously (39). Their sequences are as follows: Low (5′-TTAAATTATAAAATATTTATAATATTAATTATATATATATA AATATAATA/3SpC3/-3′) and High (5′-GCGCGGCCGGCACTGACCCGAGACTCTGAGCGGC TGCTGGAGGTGCGGAAGCGGAGGGGCGGG/3SpC3/-3′). White 96-well plates with black semi-skirting were used to maximize detection of fluorescence signal and minimize well-to-well fluorescence cross-talk (Sigma, St. Louis, MO).

*miRNA experiments.* Optimized U-dHRM miRNA reactions were performed as follows: 10 µl of total reaction volume consisting of 1× PCR buffer (Qiagen, Germantown, MD), 10 nM fluorecene (Bio-Rad, Hercules, CA), 3.5 mM total MgCl_2_ (Qiagen), 400 nM of each tag primer (IDT), 50 nM temperature calibrator sequences (IDT), 1× EvaGreen (Sigma), 200 µM dNTP (Invitrogen, Carlsbad, CA), 0.05 U/µl HotStart Taq polymerase (Qiagen), 2 µl of cDNA dilution (IDT) and ultrapure water (Quality Biological, Gaithersburg, MD), with a 15 µl of overlay of high-quality DNase-free mineral oil (Sigma). Thermocycling proceeded as follows: hold-(95°C 5 min), cycle 60 times-(95°C 30 s, 59°C 30 s), cycle 1 time-(95°C 30 s, 25°C hold). Single copy amplification by tag primers clustered around a PCR cycle threshold (Ct) of 45.

*Polymicrobial experiments.* Further optimization experiments and reduction in reaction volume combined with a previous filtration protocol (40) resulted in the final reaction conditions of 5 µl of total reaction volume consisting of 1× PCR buffer (Qiagen), 10 nM fluorecene (Bio-Rad), 3.5 mM total MgCl_2_ (Qiagen), 150 nM of each V6 primer (IDT), 50 nM temperature calibrator sequences (IDT), 1× EvaGreen (Sigma), 200 µM dNTP (Invitrogen), 0.05 U/µl AmpliTaq Gold Low DNA Taq polymerase (Invitrogen), 2 µl of genomic DNA (gDNA) dilution and ultrapure water (Quality Biological), with a 20 µl of overlay of high-quality DNase-free mineral oil (Sigma).

For each bacterial gDNA template (Table1), standard 10-fold dilutions of template gDNA in ultrapure DNase-treated water ranging from approximately 10^6^ to 0 copies per PCR reaction were run using MyIQ qPCR (Bio-Rad) machine. The thermocycling conditions that achieved reliable single copy amplification near a Ct of 45 were chosen. To ensure amplification completion, 70 cycles were run. The optimized thermocycling conditions were hold-(95°C 5 min), cycle 70 times-(95°C 30 s, 65°C 30 s, 72°C 30 s) and cycle 1 time-(95°C 30 s, 25°C hold).

### Clinical blood bottle

Clinical blood bottle isolates were grown in broth overnight at 37°C, and then DNA was extracted. Standard 10-fold dilutions of template gDNA in ultrapure DNase-treated water ranging from approximately 10^6^ to 0 copies per PCR reaction were run using MyIQ qPCR (Bio-Rad) machine and subjected to HRM for generation of database melt curves.

The blood bottle was inverted multiple times, and then 500 µl was placed in a microcentrifuge tube and briefly centrifuged for 20 s to precipitate solids. Two hundred microliters of the supernatant was used for DNA extraction. Because of the limited throughput of reactions possible with our current HRM equipment format, a preliminary dilution PCR experiment was performed to identify the appropriate digital dilution level for U-dHRM. The blood bottle extract DNA was serially diluted in 10-fold increments to 10^−^^10^. PCR was performed on each dilution in triplicate followed by HRM. The first dilution where all three reactions were negative was chosen for use in U-dHRM. Digital PCR reaction volumes were further reduced to 1 µl to reduce positives from Taq contamination and allow single bacterium detection. Reagents and final concentrations were the same as those used in the polymicrobial experiments.

### High-resolution melt (HRM)

Directly after PCR, HRM was performed on the 96-well digital plates using LightScanner (BioFire Diagnostics, Salt Lake City, UT) with a temperature range from 55°C to 95°C. Analysis was accomplished using the LightScanner software with Call-IT 2.5 small amplicon genotyping algorithm, which incorporates temperature shifting and normalization using the low and high temperature calibrators. The multi-plate analysis tool was used to load both the known database experimental curves and the unknown dHRM experimental curves into the software simultaneously. The exact algorithm settings used to normalize, temperature shift and group the known database curves are thereby directly applied to the unknown experimental dHRM curves allowing for curve identification by the software algorithm.

### Quantitation of species

For each type of positively identified melt curve grouped by the LightScanner software, the sum of matching curves was calculated along with the number of negative wells. Using these values, the concentration of each type of target molecule is calculated by Poisson statistics (41). The Poisson probability (P) of occupancy (λ) in any well, i.e. the fraction of wells having 0, 1 and so forth copies of target, is given by *P* = (λ^n^/n!) *e^(^^−^^λ)^, where *n* is the total number of wells. In terms of positive wells only, this equation becomes λ = −ln(1 − p), where p is the fraction of positive reactions. The overall average occupancy is given by the sum of the occupancy of each species λ_overall_ = λ_1_ + λ_2_ + … λ_n_. At conditions where the overall λ is low, i.e. the sample is dilute or the number of reactions is large, each reaction will contain only one starting molecule, and all species can be resolved and quantitated by U-dHRM.

## RESULTS

### Principles underlying U-dHRM

**Figure 1.**
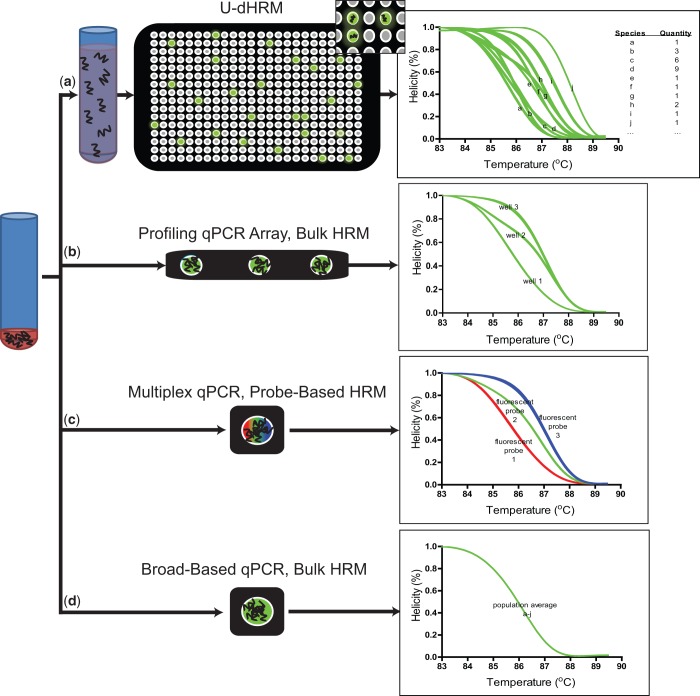
Advantages of U-dHRM over other rapid low-cost profiling methods. Exemplary representations: (**a**) U-dHRM uses a diluted sample input such that either zero or one copy of the target molecule is distributed per reaction well. Broad-based primers amplify all targets individually giving a single melt curve for each target/reaction and allowing quantification and identification of all species, including unknown or unexpected species, in the mixture. (**b**) qPCR arrays require that the sample input be divided among multiple wells resulting in multiple target molecules per well and only one primer set per well targeting an individual species; sensitivity is inversely related to the number of reactions. Low-level species may not be distributed to a well containing appropriate primers, and unknown species will not be discovered. (**c**) Multiplexed qPCR involves a single reaction containing all target molecules and multiple sets of specific primers and probes targeting individual species; single copy sensitivity is possible. However, the number of detectable target species is limited by the resolution of fluorescent probe spectra to about 4 species and unknown targets will not be discovered. (**d**) Broad-based qPCR followed by bulk HRMA can identify homogeneous target molecules, but in a heterogeneous sample cannot distinguish components of a mixture involving unknown targets.

Digitization enables HRM to accomplish both identification and quantification of many more target molecules than traditional HRM methods (Figure 1). Because U-dHRM is an extension of dPCR, the same principles apply: the distribution of digital melt curves is governed by stochasticity, and the quantification of species by U-dHRM can achieve greater precision than qPCR quantification (8).

In addition to digitization, the integration of three key techniques enable the multiplexing and accuracy achieved by the U-dHRM assay: (i) the incorporation of broad-based primers or universally ligated priming sites for unbiased amplification of all molecules of interest; (ii) shifting the burden of discriminating all amplified sequences from primers and probes to digital melt curves for specificity that relies on the inherent physical properties of the sequence flanked by conserved primer sequences; (iii) highly optimized reaction conditions that incorporate tools for normalization to enable database matching. Under these HRM conditions, single nucleotide resolution of numerous individual targets is possible. The resulting digital, temperature calibrated and stabilized melt curves are reliably sequence specific and accurately identifiable by matching to a previously generated database of temperature calibrated melt curves. Traditional bulk HRM of a heterogeneous sample cannot accomplish the same feat, as each nucleic acid sequence in the mixture will contribute to a single complex melt curve, which is impossible to decouple into individually contributing species when some of the targets in the sample are unknown, e.g. contamination (14,26).

### Practical considerations

The accuracy and reproducibility of U-dHRM melt curves relies first on optimal dPCR reaction conditions so that single copies of template are reliably amplified, primer-dimers and non-specific amplification products are averted, and the reaction is cycled to completion. This precludes false negative or erroneous melt curves in the downstream analysis. Thus, primer concentration is minimized and cycling extended during optimization. Primer specificity is also critical, as U-dHRM is highly sensitive to even single nucleotide differences in amplicon sequence, but relying on annealing temperature for control of specificity is risky owing to the inherent technical challenges of ensuring uniform heating across all reactions and the need to adapt for each primer set involved. Instead, buffer conditions were optimized by including ammonium and potassium ions, which universally stabilize specific annealing and destabilize non-specific hydrogen bonding, respectively (38). This promotes specificity across a wide range of annealing temperatures for any primers. Using universal and broad-based primers ensures that primer specificity is equivalent across all targets so that discrimination of species relies only on the sequence between the conserved priming sites.

Polymicrobial U-dHRM reactions required further optimization due to challenges associated with bacterial nucleic acid contamination. PCR reagents often contain background levels of bacterial gDNA, particularly Taq polymerases generated with recombinant DNA in bacterial cultures (32,35). Indeed, ∼1–1.5 copies of contaminating gDNA per well on average was observed in initial U-dHRM experiments. This level of background in the PCR reagents obscured digitization of the target sequences and resulted in complex multi-species melting curves within the majority of wells. Using a filtration protocol, contamination was reduced (40). All PCR reagents except Taq polymerase and target gDNA were first mixed and then filtered to remove contaminating microbial DNA. A highly purified Taq polymerase with low background DNA contamination was also used. All water used for polymicrobial experiments was DNase treated and heat inactivated. Reduction in reaction volume was also necessary to overcome this problem such that digitization could be achieved. Borrowing from microfluidic principles, reaction volumes were halved, reducing the number of contaminating molecules while maintaining the same reagent concentrations. These additional key steps made broad-based bacterial U-dHRM possible.

### U-dHRM for miRNA profiling

**Figure 2.**
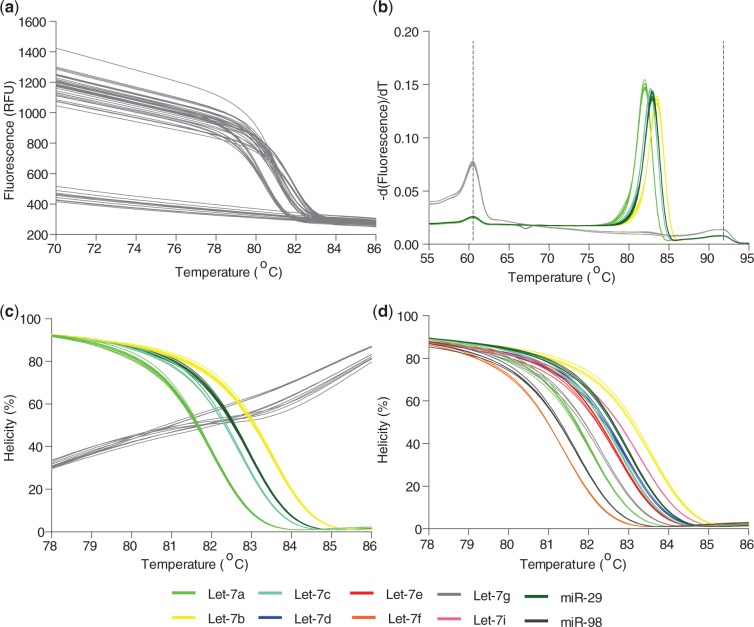
Lethal-7 and related miRNA are resolvable by HRM. (**a**) Raw HRM data, before calibration and normalization, combined from two experiments showing the loss of fluorescence in individual wells containing standard dilutions of Let-7a, b, c or miR-29 (unmixed). Flat lines are negative for amplified product. (**b**) A derivative plot of the fluorescence data from *a,* which has been temperature shifted by alignment of the temperature calibrator curve peaks, vertical dotted lines. Multiple melt curves from various dilutions of each sequence now overlap. Negative wells (gray lines) give only temperature calibrator melt curves. (**c**) Helicity plot of data from b after normalization and temperature calibration showing highly reproducible, unique melt curves for each tagged miRNA sequence and negative controls are clearly distinguishable (gray curves). (**d**) Normalized, temperature calibrated standard curves generated from 5 ten-fold dilutions across two independent experiments using each Let-7 family and related miRNA sequence from Table 1 give database references for future U-dHRM target identification.

**Figure 3.**
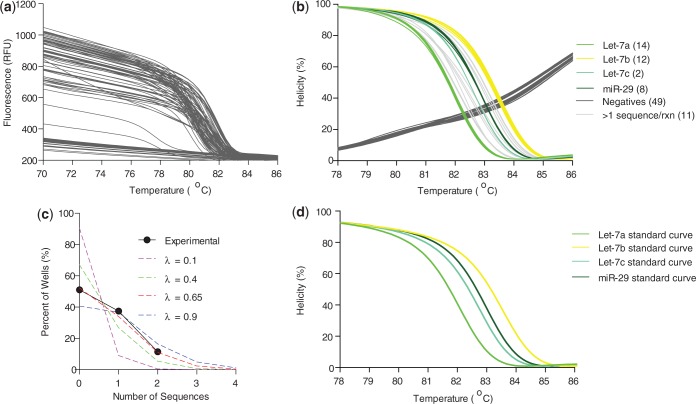
A heterogeneous mixture of Lethal-7 miRNA sequences is resolvable by U-dHRM. (**a**) Raw dHRM data showing the loss of fluorescence in each well of a 96-well plate across which a dilute mixture of Let-7a, b, c and miR-29 was dispersed. (**b**) Normalized, temperature calibrated and database matched melt curves show universally amplified, single copy detection of each of the four input miRNA (colored melt curves), detection of wells containing multiple copies of targets (light gray, non-matching curves), and amplification negative wells (dark gray melt curves). The number of copies detected is shown in parentheses next to each legend label. (**c**) Confirmation of digital detection is accomplished by enumerating and plotting each category of melt curves from graph b and comparing with the expected Poisson distributions. The experimental occupancy matched a Poisson distribution for λ = 0.65 copies/reaction. (**d**) Database curves used for identification.

Here, we demonstrate that the most difficult miRNAs to correctly identify by current microarray and qPCR methods (4,42), members of the Let-7 family differing by only 1–4 nt in sequence (Table 1, differences in bold), can be identified by U-dHRM. Likewise, miRNAs having drastically different sequences can also be identified (e.g. miR-29 and miR-98, Table 1). To accomplish unbiased universal amplification, Let-7a, b, c, d, e, f, g, i, miR-98 and miR-29 sequences were tagged for universal priming (see ‘Materials and Methods’ section). In database generation experiments, each tagged cDNA sequence was serially diluted down to the digital level, universally amplified with tag primers, and HRM was performed on each homogeneous reaction product. Figure 2A shows the raw fluorescence melt data from multiple runs of standard dilutions of Let-7a, Let-7b, Let-7c and miR-29. Wells negative for amplification are clearly distinguishable as the flat lines in Figure 2A and gray lines in Figure 2B and C. A derivative plot of the fluorescence data was generated, and alignment and normalization according to the low and high temperature calibrator sequences were performed (Figure 2B). Figure 2C shows the calibrated and normalized melt data as a percentage of the highest fluorescence, i.e. when amplicons are fully annealed in a helical structure. Optimization of reaction conditions resulted in highly reproducible melt curves for each sequence in Table 1, and these were readily distinguishable using the LightScanner’s small amplicon genotyping algorithm (Figure 2D). The melt curves in Figure 2 were collected over multiple dilutions and multiple days of experimentation, demonstrating the reliability of the optimized assay. Based on known amounts of Let-7a, b, c and miR-29, a spiked mixture was prepared, digitized and universally amplified, and U-dHRM analysis was performed. The calculated input gave a theoretical occupancy, λ_overall_ = 0.4 copies of miRNA. Figure 3 shows the results of universal U-dHRM for the mixture of miRNA. Normalized melt curves were reliably matched to a previously generated database of temperature calibrated melt curves for each miRNA (Figure 3B). From the mixture, 14 Let-7a miRNA, 12 Let-7b, 2 Let-7c and 8 miR-29 sequences were detected. Of 96 reactions, 11 were unidentified, meaning the melt curve resulted from a combination of two or more of the input sequences, and 49 reactions were negative. Digitization was confirmed by fitting these values to a Poisson distribution. Figure 3C shows the Poisson distribution for different values of λ, including that expected for λ = 0.4. Experimental quantification gave a Poisson distribution with λ_overall_ = 0.65 instead of the calculated 0.4 (Figure 3C). The overall concentration of the input mixture is then (0.65 copies/reaction)/(2 µl of input mixture/reaction) = 0.325 copies/µl miRNA. With an increased number of digital reactions, absolute quantitation of each miRNA species can be achieved (see ‘Discussion’ section).

### U-dHRM for diagnosis of polymicrobial sepsis

**Figure 4.**
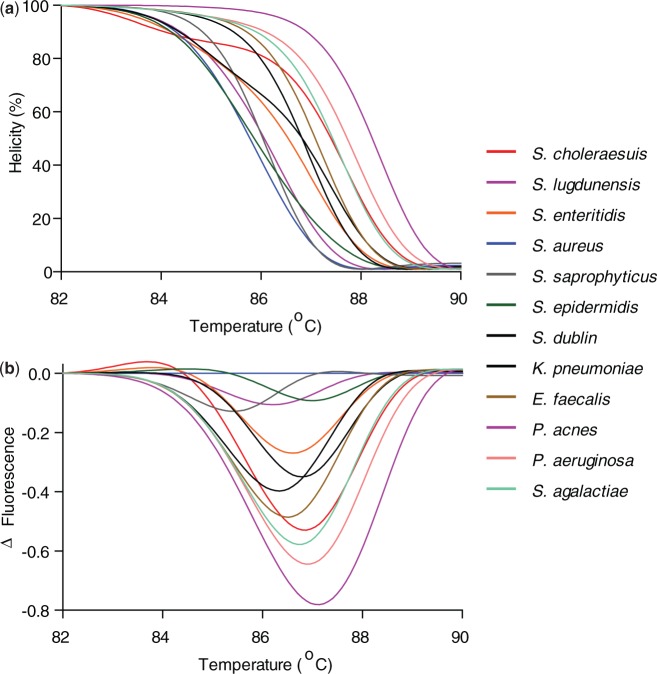
Twelve clinically relevant bacteria are resolvable by HRM. (**a**) Normalized and temperature calibrated database melt curves for each of the clinically relevant bacteria listed in Table 2 are resolvable, demonstrating the high sensitivity of HRM. (**b**) Difference curves of each bacteria using *S. aureus* as a reference. In previously work that did not include temperature calibrators or PCR optimal buffers, V6 amplicons of *S. aureus*, *S. epidermidis* and *S. saprophyticus* were not resolvable by HRM (14).

**Figure 5.**
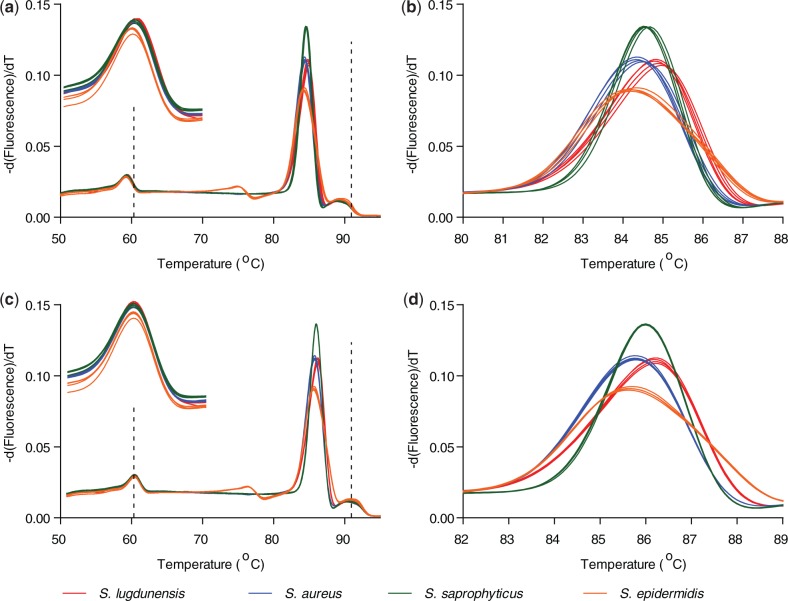
Temperature calibration and optimal PCR buffer allow highly similar *Staphylococcus species* to be resolved. (**a** and **b**) Derivative plots of HRM fluorescence data before temperature shifting; melt curves for the same species do not accurately overlap. Inset in (a) is an enlarged view of the low temperature calibrator melt curve showing slight differences in melting peak. (**c** and **d**) Derivative plots of HRM fluorescence data after temperature shifting showing improved matching and resolution. Four 10-fold dilutions of each of the four species are plotted. Dotted vertical lines show the temperatures of alignment for each of the calibrator sequences.

**Figure 6.**
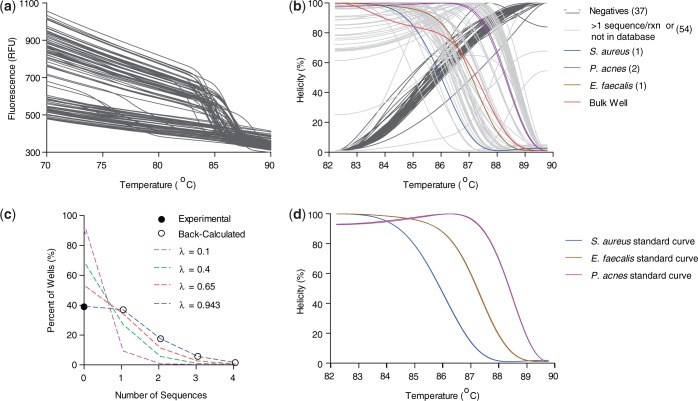
A heterogeneous mixture of bacterial gDNA is resolvable by U-dHRM. (**a**) Raw dHRM data showing the loss of fluorescence in all but one well of a 96-well plate across which a dilute mixture of *S. aureus*, *E. faecalis* and *P. acnes* was dispersed. (**b**) Normalized, temperature calibrated and database matched digital melt curves after 70 cycles of PCR show broad-based amplified, single copy detection of each of the four input bacteria (colored melt curves), detection of wells containing multiple copies of input or contaminating gDNA templates from Taq polymerase (light gray, non-matching curves), and negative wells (dark gray melt curves). The number of copies detected is shown in parentheses next to each legend label. The corresponding ‘bulk’ well (red curve) where an amount of each target gDNA equivalent to that diluted across the rest of the plate, and also including contaminating Taq gDNA, was assayed by conventional bulk HRMA. (**c**) Poisson distribution matching to the results of broad-based digital detection of polymicrobial input by U-dHRM. Enumeration of negatives allows us to calculate that an experimental occupancy of ∼0.943 was achieved. (**d**) Standard database curves used to identify target melt curves.

Here, we demonstrate that U-dHRM with broad-based bacterial 16s rDNA primers targeting the hypervariable V6 region and DNA intercalating dye can resolve each bacteria within a mixture of pathogens involved in polymicrobial sepsis and common clinical contaminants (Table 2). Melt curves were generated from standard dilutions reaching the digital level to ensure accurate curves for database creation. Calibrated and normalized helicity and difference curves of 12 clinically relevant bacteria were experimentally generated for the database (Figure 4). Highly pure and concentrated gDNA from laboratory stock organisms was used to generate standard melt curves, as any cross-contamination by other bacterial DNA would be amplified by the universal primers and contribute to changes in the melt curves. The amplicons were subsequently sequenced for identity validation (Supplementary Table 1). Difference curves of *S. aureus, S. epidermidis* and *S. saprophyticus* V6 amplicons were previously unresolvable (14), but under the currently optimized U-dHRM conditions are highly reproducible and discernible using only intercalating dye as the reporter (Figure 5). Next, a spiked mixture of *S. aureus*, *E. faecalis* and *P. acnes* was prepared, digitized, amplified by V6 broad-based primers and assessed by U-dHRM. Figure 6 contrasts a bulk HRMA experiment (red melt curve) and the U-dHRM results (all other melt curves) of the polymicrobial mixture. Input concentrations were adjusted such that the same amount of the polymicrobial gDNA was added to the bulk well as was distributed across the digital wells. Four curves were positively identified by matching to the database curves (Figure 6D). These were amplified from singular target templates. Knowing the number of negatives, 37, and total positives, 58, a Poisson calculation gives λ_overall_ = 0.94 for the reaction mixture (Figure 6C). By this calculation, ∼31 of the unidentified melt curves represent single copies of unknown gDNA templates, though not necessarily all distinct from one another. These may have originated either from the PCR reagents themselves or potentially from low level contaminants amplified by culture and carried over into the spiked gDNA input. Estimates of Taq contamination as stated by the manufacturer are ∼10 copies/µl Taq, which gives a λ_PCR contaminants_ ∼ 0.5 for the polymicrobial U-dHRM assay. The original input mixture of *S. aureus*, *E. faecalis* and *P. acnes* gDNA then has λ_input_ ∼ 0.44.

**Figure 7.**
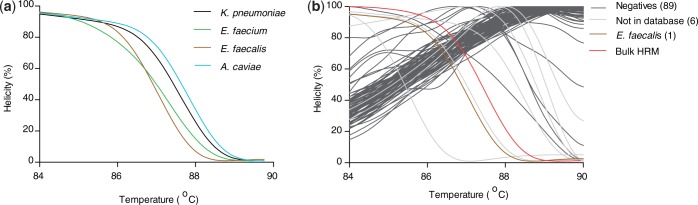
U-dHRM identifies bacteria in a clinical blood bottle. (**a**) Standard database melt curves generated from four types of bacteria isolated from the polymicrobial blood bottle. (**b**) Normalized, temperature calibrated and database matched digital melt curves after 70 cycles of PCR. Broad-based amplified, single copy detection of *E. faecalis* in the blood sample extract was achieved (brown melt curve). The number of wells containing single contaminating gDNA templates from Taq polymerase (light gray non-matching curves) is few. Negative wells (dark gray melt curves) give a calculated λ = 0.075. The number of copies detected is shown in parentheses next to each legend label. The corresponding ‘bulk’ melt curve (red curve) where an amount of DNA sample equivalent to that diluted across the rest of the plate, and also including contaminating Taq gDNA, was assayed by conventional bulk HRMA.

As further validation of the utility of U-dHRM, we next tested a clinical blood bottle from a patient identified as having polymicrobial infection with *E. faecalis*, *Enterococcus faecium*, *Aeromonas caviae* and *K. pneumoniae*. For these experiments, we relied on the Johns Hopkins Hospital clinical microbiology laboratory for culture and phenotypic identification of the bacteria in the sample as the gold standard reference. Because our database does not yet include melt curves for all clinically relevant bacteria, which numbers in the hundreds, we also acquired the blood bottle bacteria as isolates from the clinical microbiology laboratory to generate additional database curves (Figure 7A). DNA extracted from the blood bottle was then tested with U-dHRM and compared with the database organisms for matching. Figure 7B shows the resulting melt curves where *E. faecalis* was correctly identified at the single cell level. The bulk melt curve of the blood DNA extract did not match curves in the database. Reaction volume reduction to 1 µl reduced the number of non-matching contaminating DNA such that an overall λ = 0.075 was achieved. Technology that greatly increases the number of digital reactions, decreases the reaction volume and incorporates U-dHRM is needed to achieve absolute quantitation of each bacterial species (see ‘Discussion’ section).

## DISCUSSION

The absolute quantification and identification of numerous target genotypes, including discovery of unexpected or unknown species, in a heterogeneous sample using a generic reporter dye such as EvaGreen is possible by U-dHRM. This technique uses limiting dilution digitization to partition target nucleic acids across many reactions allowing discretized HRM, where each sample molecule is represented by a single and specific melt profile. Broad-based primers or universal tagging allows unbiased amplification of all nucleic acids of interest. Sample-to-answer is achieved with a single assay in a few hours. Single nucleotide resolution, single molecule sensitivity and broad-based multiplexing offer improvements to traditional microarray and qPCR profiling.

This technology can be easily expanded to identify many more microbes or miRNAs. For example, melting curve prediction software suggests that >130 bacteria could potentially be resolved (data not shown). Future work will involve additional clinical testing and database building, including organism sequence variations and comparing with clinical phenotypic differences, to build a full library of clinically relevant melt curves.

However, some foreseeable limitations exist. Although melting temperatures of the amplicons used in this study were highly repeatable, within 0.01°C, overlap of future target melt curves may occur. For example, V6 amplicons from two distinct targets can be identical in sequence, or an amplicon with an entirely different sequence may melt in way that is too similar to distinguish. Strategies to further expand the number of targets identifiable by U-dHRM include incorporating multiple broad-based primer sets and unlabeled probes or generating longer amplicons (data not shown).

In its current format, U-dHRM is also limited in its quantification ability. Unlike dPCR, U-dHRM’s quantification power requires that the number of reactions greatly outnumber the number of input molecules such that λ is low and each melt curve originates from a singular target. The sum of each type of melt curve then relates to the original concentration of individual species in the heterogeneous sample. Although the current format of U-dHRM identified single sequences in both polymicrobial and miRNA experiments (Figures 3B, 6B and 7B), absolute quantities of each sequence cannot be resolved without greatly expanding the number of reactions. More reactions will ensure an entire sample can be processed, that many individual species can be identified separately, and that the likelihood of two or more sequences occupying the same well is extremely low (Table 3). In general terms, an optimal lambda, which will give single molecule sensitivity for any application is λ < 0.0015. Therefore, the utility of U-dHRM in diagnostic and research applications will be improved by future work to increase the number and reduce the volumes of digital reactions, leading to improved resolution in the presence of contaminants, higher content, higher throughput and reduced reagent costs. High-throughput microfluidic digital droplet technologies (8,43–46) that incorporate simultaneous highly controlled heating and sensitive fluorescence detection for millions of reactions are needed. In a real heterogeneous sample where unknown sequences are expected and starting concentrations of targets may be unknown, millions of broad-based U-dHRM reactions will ensure enough dynamic range for successful single molecule detections (Table 3). Also, characterization of the contaminants in Taq and other reagents by U-dHRM database building will allow non-relevant targets to be rigorously identified and excluded from profiling analysis.

**Table 3.** Calculated dynamic range of U-dHRM detection as a function of the number of reactions

λ	Percentage of negative wells	Percentage of wells with 1 target molecule	Percentage of wells with 2 target molecules
0.001	99.90%	0.10%	0.00%
0.01	99.00%	0.99%	0.01%
0.1	90.48%	9.05%	0.45%
0.15	86.07%	12.91%	0.97%
1	36.79%	36.79%	18.39%

Total reactions	λ	Dynamic range of single molecule detection	

1000	0.001	1	
1 000 000	0.001	1.00E+03	
10 000 000	0.001	1.00E+04	
100 000 000	0.001	1.00E+05	
1 000 000	0.001	1.00E+03	
1 000 000	0.01	9.90E+03	
1 000 000	0.1	9.05E+04	
1 000 000	1	3.68E+05	

This technology has the potential to greatly impact the need for next-generation diagnostics in clinical microbiology and biomarker research. Clinical microbiology currently relies on lengthy culture-based assays to diagnose infections such as sepsis, which has a high mortality rate that continuously increases with every hour of inappropriate treatment (2,5). Generally, immediate conservative treatment with broad-spectrum intravenous antibiotic therapy is initiated without any diagnostic information leading to inaccurate and overtreatment as well as misuse of multiple antibiotics giving rise to the emergence of drug resistant pathogens (6). The ability of U-dHRM to quantify even low-level targets within hours may prove especially useful for diagnosing polymicrobial sepsis, which is associated with an even higher mortality rate than monomicrobial sepsis (2). The participation of other Gram-positive and Gram-negative bacteria, anaerobic bacteria, fungi or novel and emerging pathogens in infection can demand tailored treatments, making fast and accurate broad-based profiling to identify multiple microbes highly desirable. Likewise, the ability to quantify the heterogeneity of species present in a sample may aid clinicians in identifying true sepsis versus contamination in an otherwise ambiguous sample where some bacteria can function in either category, e.g. coagulase negative staphylococci (33).

Biomarker research may also significantly benefit from this technology. Recently, the expression patterns of circulating miRNAs have proven useful as diagnostic and prognostic biomarkers of various diseases (47) including prediction of mortality from sepsis (19). However, the need for high-throughput, genome-wide, accurate and quantitative miRNA profiling that allows discovery of new sequences has not been met by any singular technology (4). Hybridization-based assays used for genome-wide high-throughput miRNA profiling often give false-positive results in identifying miRNA with single nucleotide differences due to the conflict between the need for stringent annealing conditions and the drastic differences in annealing temperature of the short miRNA sequences. This is particularly problematic for distinguishing families of miRNA such as Let-7, for which each member plays an individual role in numerous biological functions (48). qPCR is currently considered the ‘gold standard’ for quantitation and detection of miRNA, but the bias associated with qPCR (Figure 1) and rapid increase in the number of miRNA renders it inefficient on a genomic scale (49). NGS is the choice for discovery, but can also be influenced by sequencing errors. The potential of U-dHRM to accomplish all of these tasks in one assay may help to increase the interpretability and reproducibility of findings in this young field (4,7).

The ultimate utility of the U-dHRM assay lies in its ability to discretely yet universally analyze the components of a heterogeneous nucleic acid sample while remaining compatible with other PCR, HRM and NGS technologies. It should therefore prove accessible and useful for research involving various nucleic acid molecules and sample types.

## SUPPLEMENTARY DATA

Supplementary Data are available at NAR Online.

## FUNDING

E.W., ‘Al’ Thrasher Research Award, National Science Foundation [1159771 and 1033744]; Mid Atlantic Regional Center of Excellence for Biodefense and Emerging Infectious Diseases NIAID/NIH [U54 AI057168], and National Institutes of Health [R01CA15305, U54EB007958 and AI068613-01]; Burroughs Wellcome Fund Career Award at the Scientific Interface (to S.F.). Funding for open access charge: National Science Foundation [1159771].

*Conflict of interest statement*. None declared.

## Supplementary Material

Supplementary Data
