# Supplementary material for: Universal digital high-resolution melt: a novel approach to broad-based profiling of heterogeneous biological samples
Source: Nucleic Acids Res. 2013 Aug 9;41(18):e175. doi: 10.1093/nar/gkt684 (PMC3794612; doi:10.1093/nar/gkt684)
Supplement: Supplementary Data [file supp_41_18_e175__index.html]

Universal digital high-resolution melt: a novel approach to broad-based profiling of heterogeneous biological samples — Universal digital high-resolution melt: a novel approach to broad-based profiling of heterogeneous biological samples — Supplementary Data 

# Universal digital high-resolution melt: a novel approach to broad-based profiling of heterogeneous biological samples

## Supplementary Data

files

**Files in this Data Supplement:**

- Supplementary Data - pdf file
