# Supplementary material for: Universal digital high-resolution melt: a novel approach to broad-based profiling of heterogeneous biological samples
Source: Nucleic Acids Res. 2013 Aug 9;41(18):e175. doi: 10.1093/nar/gkt684 (PMC3794612; doi:10.1093/nar/gkt684)
Supplement: Supplementary Data [file supp_gkt684_nar-00887-met-f-2013-File012.pdf]

Supplementary Table 1. Sequencing results for organisms used in U-dHRM polymicrobial experiments.

| Organism | V6 Sequencing Result |
|---|---|
| *S. lugdunensis* | aatcttgacatcctttgaccgctctagagatagagttttccccttcgggggacaaagtgacaggtggtgcatggatgtcgtcagctaa |
| *S. aureus* | gacatcctttgacaactctagagatagagccttccccttcgggggacaaagtgacaggtggtgcatggatgtcgtcagctaa |
| *S. saprophyticus* | tgacatcctttgacaactctagagatagagccttccccttcgggggacaaagtgacaggtggtgcatggatgtcgtcagctaa |
| *K. pneumoniae* | ccacagaactttccagagatggattggtgccttcgggaactgtgagacaggtgctgcatggctgtcgtcagcta |
